# The Effects of Sex Hormonal Fluctuations during Menstrual Cycle on Cortical Excitability and Manual Dexterity (a Pilot Study)

**DOI:** 10.1371/journal.pone.0136081

**Published:** 2015-08-26

**Authors:** Maryam Zoghi, Bita Vaseghi, Andisheh Bastani, Shapour Jaberzadeh, Mary P. Galea

**Affiliations:** 1 Department of Medicine (Royal Melbourne Hospital), The University of Melbourne, Parkville, Melbourne, Australia; 2 School of Primary Health Care, Faculty of Medicine, Nursing and Health Sciences, Monash University, Frankston, Melbourne, Australia; John Hopkins University School of Medicine, UNITED STATES

## Abstract

**Aim:**

To investigate whether hormonal fluctuations during the menstrual cycle affect corticospinal excitability, intracortical inhibition (ICI) or facilitation (ICF) in primary motor cortex, and also whether the hormonal fluctuations have any effect on manual dexterity in neurologically intact women.

**Materials and Methods:**

Twenty volunteers (10 Female, 10 Male) were included in this study. The levels of progesterone and estradiol were measured from saliva during the women’s menstrual follicular, ovulation and mid-luteal phases. Motor evoked potentials were recorded from the right first dorsal interosseous muscle. Single and paired-pulse Transcranial Magnetic Stimulation (TMS) were delivered in a block of 20 stimuli. With paired-pulse technique, 3ms and 10ms inter-stimulus intervals were used to assess ICI and ICF, respectively. The Grooved Pegboard Test (GPT) was completed in each session before the TMS assessments. Male participants were tested at similar time intervals as female participants.

**Results:**

Mixed design ANOVA revealed that GPT score in female participants was significantly lower at the mid-luteal phase compared to the ovulation phase (p = 0.017). However, it was not correlated with progesterone or estrogen fluctuations during the menstrual cycle. The results also showed that the effect of phase, sex and the interaction of phase by sex for resting motor threshold, ICI or ICF were not significant (p > 0.05).

**Conclusion:**

Manual dexterity performance fluctuates during the menstrual cycle in neurologically intact women, which might be due to the balance of the neuromodulatory effects of P4 and E2 in the motor cortex during different phases.

## Introduction

Female sex hormones have significant impact on brain functions [1–5]. Progesterone (P4) increases cortical excitability [6–9] and seizure threshold [10, 11] and reduces saccadic eye movement velocity [12] while estradiol (E2) decreases cortical excitability and seizure threshold [11, 13] and improves cerebellar motor learning [14]. It has been shown that natural cyclic fluctuations of P4 and E2 during the menstrual cycle could influence exacerbation of seizures in women with epilepsy [15, 16]. A positive correlation was shown between seizure susceptibility and the E2-to-P4 ratio, peaking before menstruation and ovulation and declining during the mid-luteal phase [16, 17]. It has been suggested that sex hormones can affect brain excitability by modulating the activity of inhibitory and excitatory neurotransmitter receptors and ion channels [18–20].

γ-aminobutyric acid (GABA) is the most important inhibitory neurotransmitter in the brain [21]. It is estimated that 30% of the synapses in the mammalian cerebral cortex [22] are GABAergic and GABA-mediated inhibition is crucial in the modulation of cortical excitability [23]. On the other hand glutamate is the most important excitatory neurotransmitter in the brain [21]. P4 binds to a site on GABA_A_receptor which is a ligand-gated chloride channel, and allows the influx of chloride, resulting in membrane hyperpolarization and a decrease in cortical excitability [24]. E2, on the other hand, decreases the firing threshold of a neuron by increasing N-methyl-D-aspartate (NMDA)-mediated glutamate receptor activity and therefore increases cortical excitability [25]. E2 also binds the GABA_A_ receptor and alters chloride conductance, and therefore reduces GABA-mediated inhibition [26]. In fact, by inhibiting GABAergic inhibition, E2 activates pyramidal neurons [26]. P4 has the opposite effect. It enhances GABAergic inhibition, reduces glutamate excitation and inhibits pyramidal neurons [27].

### The effects of hormonal fluctuations on manual dexterity

Several studies investigated the effects of hormonal fluctuations on manual dexterity [28–35]. However, different manual tasks were used (Purdue Pegboard Test [28, 29, 31, 33], Grooved Pegboard Test (GPT) [32, 34, 35] or O'Connor Finger Dexterity Test [30]) and the results were controversial. Simic et al. (2010) reported that the fine motor task (O'Connor Finger Dexterity Test) improved in the mid-luteal phase [30]. Hampson et al. (1990) also reported that manual skill (using the Purdue Pegboard Test) improved in the late follicular phase compared with the menstrual phase [29]. However, some other studies that used the Purdue Pegboard test did not show similar changes throughout the menstrual cycle [28, 31, 33]. This controversy can be seen in the other studies that used the GPT to assess manual dexterity. Although Maki et al. (2002) reported that the GPT score improved in the mid-luteal phase compared to the follicular phase [32], Keenan et al. (1992, 1995) in two studies reported that the GPT score did not change throughout the menstrual cycle [34, 35]. Therefore, still there is no consistent result in this regard and further investigations in this area are warranted.

### Grooved Pegboard Test

The GPT is one of the assessment tools that have been used for assessing manual dexterity [36, 37]. The test kit consists 25 pegs and a board with 25 holes. Each hole has a radial ridge and they are positioned on the board with different directions. During the test, participants pick up the pegs one by one and place them in the holes as fast as they can. Since each hole has a ridge on one side with different directions, participants need to rotate their fingers to be able to fit each peg in each hole. This rotatory component makes this test more difficult than the Purdue Pegboard Test as a manual dexterity task [36, 37]. This extra rotatory component increases independent finger movements during this task, which in turn increases the cortical control (GABAergic involvement, see below) of this task. This test was used to assess manual dexterity in this study.

### Cortical control of manual dexterity

The complexity of the cortical control of the manual dexterity can be reflected from the largest parts that have been devoted to hands in the motor cortex. Many studies have been conducted so far in animals and humans to clarify different aspects of the cortical control of finger movements (see Porter and Lemon, 1993 for review) [38–40].

It has been established that the direct cortico-motoneuronal (CM) connections are necessary for controlling manual dexterity in humans [40]. The functions of these CM connections are influenced by intraneuronal circuits in the motor cortex [41–44]. These intracortical circuits can be inhibitory (ICI) or facilitatory (ICF). ICI circuits use GABA as their primary neurotransmitter [45]. It has been suggested that GABAergic inhibitory neurons assist in the selective activation of muscles by letting the required muscles to be active and keeping the rest of the muscles relaxed during the task [44, 46]. It has been shown that these circuits are abnormal in some movement disorders like task-specific focal dystonia [47], in which there is involuntary overflow of the descending motor commands to the muscles that are required to remain relaxed during a specific task. The function of ICI and ICF circuits can be assessed by Transcranial Magnetic Stimulation (TMS).

### Transcranial Magnetic Stimulation and assessing intracortical inhibitory and facilitatory circuits

Kujirai et al. (1993) showed that ICI and ICF circuits can be studied non-invasively in humans using paired-pulse TMS [48]. In this method, TMS that is subthreshold for a motor response activates these circuits and reduces or increases the size of the motor evoked potential (MEP) elicited by a supra-threshold test TMS pulse delivered up to 1–5ms or 8–15ms later respectively [48].

It is believed that the inhibition of corticospinal neurons is shown by paired TMS is cortical in origin [48], and reflects the activity of a subset of intracortical GABAergic interneurons in primary motor cortex (M1) [49, 50]. Later observations of the effects of various drugs support the proposition that MEP suppression with the paired-pulse technique is produced by GABAergic action within the motor cortex [51–53]. ICF, on the other hand is affected by both NMDA [54, 55] and GABA_A_ receptor agents [56, 57]. They suggested that since ICF and ICI have very similar pharmacological profiles, the inhibition that is reflected by ICI contributes to the net facilitation that is reflected by ICF [57].

Since E2 and P4 can affect the glutamergic and GABAergic functions in the brain, it is reasonable to speculate that fluctuations of these hormones during the menstrual cycle might affect the controlling functions of ICF and ICI circuits on CM cells that are involved in manual dexterity performance.

Although a considerable body of research has demonstrated the effects of hormonal fluctuations on cortical excitability or manual dexterity, most of these studies investigated the effects of sex hormonal fluctuations on cortical excitability or manual dexterity separately. There is limited knowledge regarding whether changes in cortical excitability are correlated with the changes in manual dexterity or not. In addition, a few studies have reported the effects of hormonal fluctuations on cortical excitability using TMS [6–8]. In these studies, the analysis methods that have been used do not fully characterise cortical inhibition.

This study was designed to investigate whether hormonal fluctuations (E2 and P4) during the menstrual cycle alter ICI and ICF in the M1 and whether hormonal fluctuations have any effect on performing a manual dexterity task (Grooved Pegboard Test, GPT) in healthy women. Another aim of this study was to investigate whether these changes (if any) are correlated.

We hypothesised that the level of ICI in M1increases in association with P4 levels during the mid-luteal phase. We also hypothesised that manual dexterity during GPT would improve during the mid-luteal phase compared to the ovulation phase in healthy women, and that this improvement would be correlated with the level of ICI in M1.

## Experimental Procedure

### Materials and Methods

#### Subjects

Twenty healthy volunteers (10 F, 10 M) participated in this study. Inclusion criteria were: 1–20–35 years of age; 2- Being strongly right-handed (Median Laterality Quotient of ≥ 0.85, calculated from the Edinburgh Handedness Inventory questionnaire). 3- Having a regular menstrual cycle of 28–30 days during the last six cycles (for women). Exclusion criteria (for women) were: 1- Fluctuation in cycle length of more than ± 1 day; 2- Use of hormonal contraceptive methods; 3- Being pregnant; 4- Having lactation and 5- History of ovariectomy. Exclusion criteria (for both sexes) were: 1- Having endocrinological abnormalities (such as hyperthyroidism or hyperandrogenaemia); 2- Having any musculoskeletal injuries in upper limbs; 3- Having any neurological conditions.

All participants gave their written informed consent before the assessments were carried out. All procedures used conformed to the Declaration of Helsinki, and the protocol was approved by the Human Research Ethics Committees at The University of Melbourne and Monash University.

#### Measuring hormone levels (for women)

Previous literature comparing parallel serum and saliva samples highlighted the advantages of using saliva over serum [58, 59]. Collecting a saliva sample is simple, non-invasive and it does not produce any stress for patients. In addition, it can be collected at home and be sent off to the laboratory by mail. Several studies have shown highly significant correlations between salivary and serum total free steroid concentrations with correlation coefficients greater than 0.8 [60–66].

The level of Estrone (E1), E2 and P4 were measured by an enzyme immunoassay technique from saliva with a sensitivity of 5pg/mL during the following phases of a normal menstrual cycle: 1) follicular phase (days 0 to 9, when both E2 and P4 are low); 2) ovulation phase (days 12–15, when E2 is increasing but P4 remains low) and 3) in the mid-luteal phase (days 18 to 24, when both E2 and P4 are high). Since all our female participants had regular menstrual cycles, we monitored the menstrual cycle using the day count method by counting forward 12 to 15 days after the onset of menses. To confirm whether they were in their ovulation phase before testing, they were asked about the type of their cervical fluid. They all confirmed that they were having “egg white” type of cervical fluid during ovulation phase at the time of assessment.

Three saliva samples were collected from each female participant throughout the study. These samples were labeled with the participant’s code number and stored in a freezer until all testing was completed for that participant and then were dispatched to the laboratory for analysis. The order of the three phases was randomly chosen for each participant. Participants were asked to avoid caffeine consumption for two hours prior to testing. To reduce the effect of circadian fluctuations in hormone levels, all investigations were performed between 9:00 a.m. and 12:00 p.m.[67].

Healthy male participants were recruited to this study as a control group. They were tested at similar intervals as female participants.

#### Enzyme immunoassay techniques

After saliva collection, samples had to be frozen at -20°C. On the day of assay, samples were thawed, and centrifuged to separate the mucins and other particulate matter which could lead to falsely elevated results. Samples were at room temperature before being added to the assay plate.

To measure P4 level in saliva samples, the procedure was as follows. All reagents (Microtiter wells, Standard, Control, Enzyme conjugate, Substrate, Stop and Wash solutions) were brought to room temperature and mixed without foaming. The desired number of coated strips (with polyclonal antibody) was secured in a holder. 100 μl of each P4 standards and controls and 100 μl of each sample were dispensed into appropriate and selected wells. Then, 200 μl of enzyme conjugate were dispensed into each sample and standard wells and the plate was mixed thoroughly for 10 seconds and incubated for 60 minutes at room temperature. Following that, the contents of the wells were briskly shaken out and the wells rinsed 3 times with diluted wash solution. After this the inverted wells were stroked sharply on absorbent paper towel to remove residual droplets. Then, 200 μl of the substrate solution was added to each well and incubated for 15 minutes at room temperature and the reaction was stopped by adding 100 μl of stop solution to each well. Finally, the absorbance of each well was determined at 450±10 nm and the wells were read within 10 minutes.

To measure E1 level in saliva samples, the procedure was as follows. All reagents were brought to room temperature and mixed before use. Plates were also brought to room temperature and prepared for use with non-specific binding (NSB) wells. The tube was prepared with 12 mL of E1 assay diluent for conjugate dilution. 1X wash buffer was prepared and E1 standard was serially diluted. Following that, 100 μL of standards, controls, and unknowns were pipetted into appropriate wells and 100 μL of E1 assay diluent were dispensed into zero and NSB wells. Then a 1:160 dilution of conjugate (75 μL into 12 mL E1 assay diluent) were made, mixed, and immediately pipetted 100 μL of it to into each well. An adhesive cover was placed over the plate which was incubated at room temperature for 3 hours mixing constantly at 500 rotations per minute (rpm). Next, the plate was washed 4 times with 1X wash buffer, blotted and 200 μL tetramethylbenzidine solutions was added to each well. The plate was mixed for 5 minutes at 500 rpm and incubated in the dark at room temperature for an additional 25 minutes. 50 μL of stop solution was added to each well and mixed for 3 minutes at 500 rpm. At the end, the plate bottom was wiped clean and read within 10 minutes of adding the stop solution.

To measure E2 level in saliva samples, the procedure was as follows. All procedures were identical to E1 immunoassay except that a 1:800 dilution of conjugate (15 μL into 12 mL E2 assay diluent) was made, mixed, and 100 μL immediately pipetted into each well. After placement of an adhesive cover over the plate, it was mixed on a rotator for 5 minutes at 500 rpm, and was then incubated at room temperature for an additional 115 minutes. The remainder of the process was similar to the E1 immunoassay.

#### Assessing cortical excitability with transcranial magnetic stimulation

TMS was delivered with two Magstim 200 stimulators (Magstim Company Limited, UK) connected with a Bistim module (Magstim Company Limited, UK) to allow the output of both

stimulators to be discharged through the same figure of eight coil. The magnetic coil was placed over the left hemisphere (cortex), contralateral to the target muscle. The orientation of the coil was set at an angle 45° to the midline and tangential to the scalp. A scalp site optimal for evoking an MEP at rest in the first dorsal interosseous (FDI) muscle of the right hand was located and marked as a reference [68]. The coil position and orientation were constantly checked during the experiment to ensure that no changes occurred.

Participants were seated upright and comfortable with their head and neck supported by a headrest. TMS thresholds were assessed for FDI at rest (resting motor threshold, RT). MEP threshold was tested in steps of 2% maximum stimulator output, and defined as the lowest intensity for which three of five successive MEPs exceed 50 μV (rest) peak-to-peak amplitude. Test TMS intensity was adjusted to produce a test MEP in FDI at rest of about 1mV amplitude. Conditioning TMS intensity was adjusted to 0.8 x RT for each participant with 3 and 10ms ISI. Single or paired-pulse TMS was delivered randomly in a block of 20 stimuli (10s interval between stimuli) with participants at rest with each ISI (60 trials for each participant at each session).

MEP areas were quantified off-line from the digitised averages of rectified EMG for conditioned and unconditioned stimuli in each block by using a custom designed macro in Powerlab 8/30 software. The size of the conditioned MEP was expressed as a percentage of the unconditioned test MEP to assess the effectiveness of ICI or ICF.

#### Electromyography (EMG) recording

Participants were seated in an adjustable podiatry chair with their forearm pronated and the wrist joint in a neutral position, resting on the armrest of the chair. To ensure good surface contact and reduce skin resistance, a standard skin preparation procedure of cleaning and abrading was performed for each site of electrode placement. MEP responses were recorded from the right FDI, using pre-gelled self-adhesive bipolar Ag/AgCl disposable surfaces electrodes with an inter-electrode distance of 3 cm, measured from the centre of the electrodes. The location of the FDI muscle was determined based on anatomical landmarks [69] and also observation of muscle contraction in the testing position (index finger abduction). The accuracy of EMG electrode placement was verified by monitoring online EMG activity during maximal contraction of FDI. The ground electrode was placed ipsilaterally on the styloid process of the ulnar bone [70]. Then, the electrodes were secured by tape. All raw EMG signals were band pass filtered (10–1000 Hz), amplified (×1000) and sampled at 2000 Hz and were collected on a PC running commercially available software (Chart software, ADInstruments, Australia) via a laboratory analogue-digital interface (PowerLab 8/30, ADInstruments, Australia).

#### Assessment of fine motor skills with the Grooved Pegboard Test

Female participants completed the Grooved Pegboard Test with their right hand during their menstrual cycle (three times at each session after TMS assessment). Male participants went through the same assessments with similar time intervals. Participants inserted all the pegs to the holes as fast as they could with their right hand. The time required to insert all the pegs was recorded.”

## Data Analysis

Repeated-measures ANOVA was used to assess the amount of hormonal fluctuation during the menstrual cycle in female participants. A mixed design ANOVA was also used to assess the effects of Sex (Female, Male) as a between-subject variable and Menstrual phase/Time interval as a within subject variable on the relative size of the conditioned MEP and GPT score. A significance level of p < 0.05 was adopted for all comparisons. Post-hoc tests (Tukey’s test and Student’s t-tests with Bonferroni correction) were performed where indicated. Means are reported ±SEM unless otherwise stated.

## Results

Twenty participants (10 F, 10 M) were tested. One male participant was not able to finish all three assessments and his data were excluded. The hormonal profiles of one female participant were not in the normal range and therefore her data were excluded. The data of 9M and 9F with a mean (±SD) age of 24.9 ± 5.1 and 26.1 ± 5.6 years were analyzed. Table 1 shows the descriptive data for hormonal levels in female group during menstrual cycle.

**Table 1:** The descriptive data for hormonal levels in female group during menstrual cycle.

Menstrual cycle phase	Estrone (E1) (pmol/L)	Estradiol (E2) (pmol/L)	Progesterone (P4) (pmol/L)
Follicular	79 ± 12.02	7.89 ± 0.89	266.33 ± 28.25
Ovulation	95.89 ± 13.01	9.78 ± 1.39	382.5 ± 61.7
Mid-luteal	83.78 ± 9.62	11.55 ± 1.82	594 ± 112.64

pmol/L: picomoles per liter

**Fig 1:**
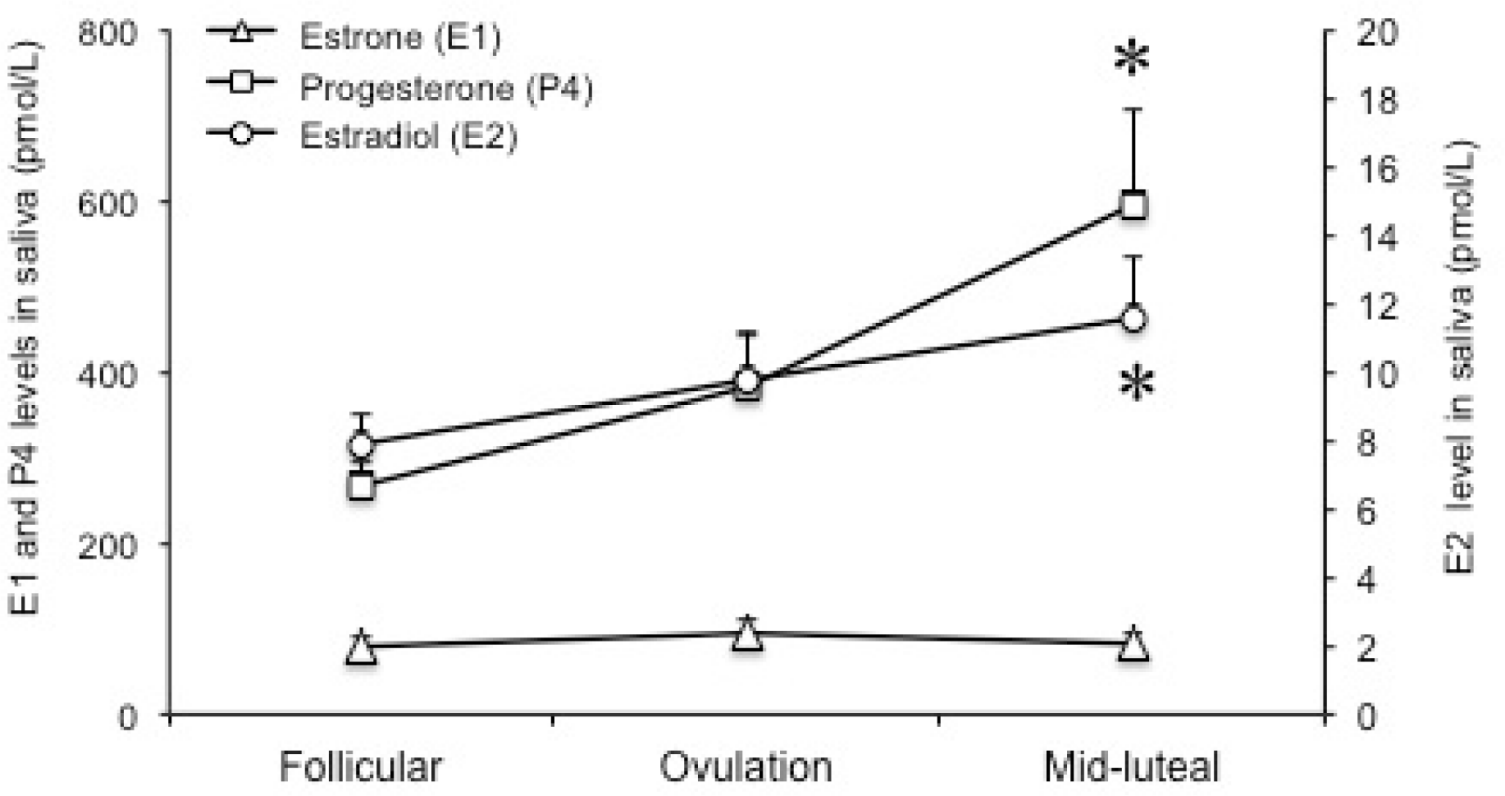
Changing hormonal levels during menstrual cycle. P4 and E2 levels were significantly higher at the mid-luteal phase compared to the follicular phase. *: p < 0.05.

The phase effect on the mean P4 level was significant [F (2,16) = 8.1, p = 0.004]. Post hoc testing showed that P4 levels increased significantly at the mid-luteal phase compared to the follicular phase (p = 0.009) (Fig 1). The mean E2 level increased from 7.8 ± 0.83 pmol/L at the follicular phase to 9.7 ± 1.31 pmol/L at the ovulation phase and then to 11.5 ± 1.71 pmol/L at the mid-luteal phase. The phase effect on the E2 levels was significant [F (2,16) = 4.9, p = 0.022]. Post hoc testing showed that E2 levels increased significantly at the mid-luteal (p = 0.017) phase compared to the follicular phase (Fig 1). The phase effect on the mean estrone level (E1) however, was not significant (p > 0.05).

**Fig 2:**
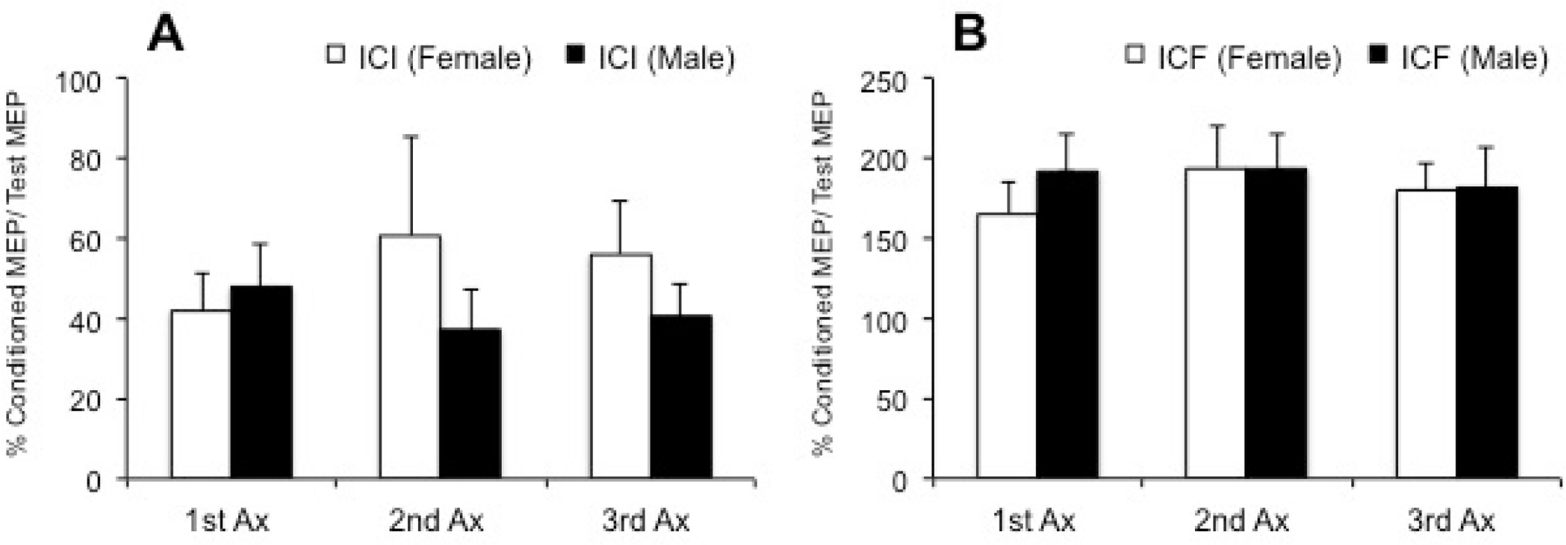
The effects of hormonal changes in cortical excitability. A) ICI changes during three Ax sessions in men and women. B) ICF changes during three Ax sessions in men and women. 1st, 2nd and 3rd Ax for men is equivalent to follicular, ovulation and mid-luteal phases respectively for female participants. *: p < 0.05. MEP: Motor evoked potential; ICI: intracortical inhibition; ICF: intracortical facilitation; Ax: assessment.

Mixed design ANOVA showed that the effects of phase, sex and the interaction of phase by sex for RT, ICI or ICF were not significant (p > 0.05). Mean ICI and ICF levels in female and male participants at three different time intervals are presented in Fig 2.

**Fig 3:**
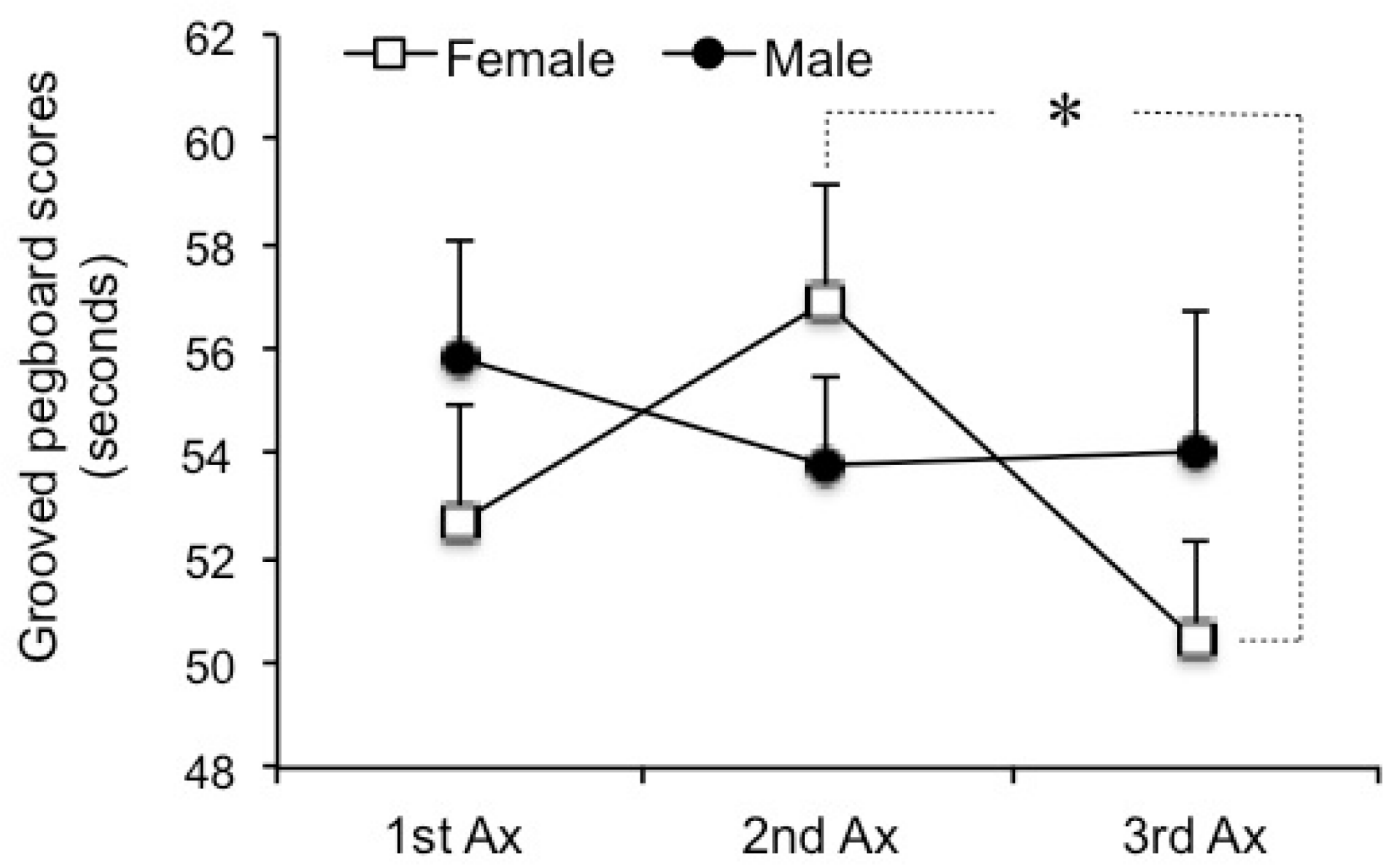
Sex differences in GPT scores. GPT score was significantly lower at the 3rd Ax (mid-luteal phase) compared to the 2nd Ax (ovulation phase) in female participants. GPT scores at the three assessment sessions remained unchanged for men. *: p < 0.05. GPT: Grooved Pegboard Test; Ax: assessment.

On the other hand the interaction between phase and sex and GPT score was significant [F (2,32) = 5.37, p = 0.01]. Further analysis showed that the GPT score in female participants was significantly lower at the mid-luteal phase compared to the ovulation phase (p = 0.017). However, the GPT score in male participants remained unchanged at each of the three assessments (p > 0.05) (Fig 3). There was no correlation between P4 or E2 levels with GPT score at the mid-luteal phase (p < 0.05).

## Discussion

In the present study, sex hormones (E2 and P4) were measured in saliva, in contrast to previous studies measuring these in blood serum. It has been shown that salivary and serum measures of free steroid concentrations are highly correlated (correlation coefficients > 0.8) [60–66].

### Changes of ICI and ICF during the menstrual cycle

We hypothesised that the level of ICI in M1increases during the mid-luteal phase. Our first hypothesis was not supported by the results of the present study. We showed that ICI levels remained unchanged throughout the menstrual cycle, which is in agreement with the results of two previous studies [7, 8] and in contrast with another study [6]. Smith et al. (1999) showed that ICI was significantly higher during the mid-luteal phase compared to the follicular phase [6]. However, in 2002, the same group reported that ICI levels were found to be similar at follicular and mid-luteal phases [7].

To measure the ICI index, Smith et al.^6,7^ calculated the ratio of the conditioned MEP *amplitude* (mV) to the unconditioned MEP *amplitude* (mV), which was different from the method used in the present study. In the present study the areas of the MEP responses (mV.S) were measured and the ratio of the conditioned MEP area to the unconditioned MEP area was calculated as the ICI index. This ratio provides a better representation of the ICI effects on MEP responses with the areas of the MEP responses compared to peak to peak MEP *amplitudes* as we measured all the effects compared to some part of the effects.

Smith et al. (2002) also showed that during the ovulation phase, ICI was significantly decreased compared to the follicular phase, which was in contrast with the present study [7]. One reason that might explain this discrepancy during the ovulation phase is that this group assessed the ICI effects at different ISIs (2–5ms) and they combined the results from all these ISIs for the final analysis. Therefore their final results were contaminated with the effects of 2 ms ISI which are different from 3–5 ms ISIs [44]. Hanajima et al. (2003) compared the effects of paired-pulse TMS at 1, 2 and 3–5 ms [71] and showed that ICI produced by paired-pulse TMS has different physiological characteristics at different ISIs [71]. They reported that at an ISI of 2 ms, none of the descending volleys in the corticospinal tract were inhibited. They suggested that only ISIs of 3–5 ms should be used for estimating the function of the GABAergic inhibitory system in the motor cortex with the paired-pulse TMS technique [71].

The present study also showed that the ICF level remained unchanged throughout the menstrual cycle, which is consistent with the results of previous studies [6–8]. It has been shown that ICF is decreased by GABA_A_ mediated inhibition [50, 72] and the inhibitory and facilitatory effects summate at the cortical level [51, 73, 74]. However, in the present study, the level of ICF did not change during menstrual cycle. During ovulation phase, the E2 level increased while the P4 level remained unchanged. Since E2 can increase cortical excitability by increasing glutamate receptor activity [25] and also by reducing GABA-mediated inhibition [26], ICF could increase during the ovulation phase. However, this was not the case in the present study. Although a trend of increasing E2 levels was observed during the ovulation phase, this did not reach significance compared to the follicular phase. It is possible that we may not have assessed some of the female participants during their ovulation phase despite applying two methods of detecting this phase (day count and cervical mucus methods). Therefore, the E2 level changes during the time of our assessment, were insufficient to change the ICF level significantly. In future studies, ICF changes during ovulation phase could be explored using a more sophisticated method of detecting the ovulation phase in female participants and also having a larger sample size.

### Changes in manual dexterity during the menstrual cycle

Our second hypothesis regarding improvement of manual dexterity at the mid-luteal phase compared to the ovulation phase was supported by the results of this study as well as that of a previous study [32]. Some studies however, reported that manual dexterity remained unchanged during follicular and luteal phases [34, 35]. It is important to note that in these studies manual dexterity was not assessed at the ovulation phase (late follicular phase). In fact manual dexterity was assessed at the early follicular phase (when both E2 and P4 were at their lowest levels) and remained unchanged compared to the mid-luteal phase (when both E2 and P4 levels were high). In this case their results are consistent with the results of the present study (comparing to follicular phase rather than ovulation phase).

A functional neuroimaging study reported that the functional cerebral organisation of fine motor coordination is sex-sensitive and might be modulated by sex hormones [75]. The physiological basis for the results of the present study seems to be related to the neuromodulatory properties of P4 and E2 on neurotransmission in the motor cortex in women.

In primates the direct CM connections are essential for manual dexterity [38, 76] and their function is influenced by GABAergic interneurons in the motor cortex [41–44]. These GABAergic neurons are involved in masking excitatory connections between inappropriate CM connections and therefore enhance the synaptic efficiency in specific intracortical pathways. This is consistent with observations that application of drugs which alter GABA neurotransmission in the motor cortex in the monkey degrades the independence of finger movements [43], disrupts task-specificity of pyramidal neurons [41] and produces excessive co-activation of agonist and antagonist muscles.

It has been shown repeatedly that P4 facilitates GABAergic transmission by acting on GABA_A_ receptors [77–80], which in turn can result in inhibitory effects on neuronal function. In addition P4 can suppress the excitatory glutamate response [81], whereas E2 suppresses GABAergic synaptic inputs [82] and therefore the inhibitory effects on neuronal function [82].

During ovulation, E2 level reaches its highest level. Our results showed that, during ovulation, female participants could not perform the GPT as well as during the mid-luteal phase. We also showed that there was no correlation between the P4 level and the GPT scores. One possible reason for this observation might be due to the disturbance of the ratio of E2 to P4 in this phase compared to the mid-luteal phase, rather than the level of P4 alone. However, we have to take into consideration the possibility that we may not have assessed all the female participants during their ovulation phase, despite of applying both methods of detecting the ovulation phase (day count and cervical mucus methods).

During the ovulation phase, increasing the E2 level could suppress the GABAergic synaptic inputs to CM connections, which in turn could affect their controlling effects during a manual dexterity task. Therefore, women are not able to perform the task as well as before. However, when the P4 level increases during the luteal phase, it will counteract the E2 effects by facilitating the GABAergic transmission and therefore, their performance will return to the previous level. In fact, increasing the P4 level in the mid-luteal phase will bring the ratio of E2 to P4 back to the ratio during the follicular phase, and therefore the balance of neuromodulatory effects of P4 and E2 in the motor cortex will be similar to the follicular phase.

## Limitations

This study has some limitations. Although powered to detect differences in some variables, our findings are based on a small sample of 20 individuals (10M, 10F). We were aiming to assess all female participants during their ovulation time, however, there is a possibility that we may not have assessed all the female participants during their ovulation phase despite applying both methods of detecting the ovulation phase (day count and cervical mucus methods).

## Conclusion

Manual dexterity performance fluctuates during the menstrual cycle in neurologically intact women, which might be due to the balance of the neuromodulatory effects of P4 and E2 in the motor cortex during different phases.
